# Comparison of efficacy and adverse events by treatment cycles of nivolumab and ipilimumab in Japanese melanoma patients: A single‐center, retrospective study

**DOI:** 10.1111/1346-8138.17672

**Published:** 2025-03-05

**Authors:** Ken Horisaki, Shusuke Yoshikawa, Wataru Omata, Arata Tsutsumida, Yoshio Kiyohara

**Affiliations:** ^1^ Department of Dermatology Shizuoka Cancer Center Shizuoka Japan; ^2^ Department of Dermatology Nagoya University Graduate School of Medicine Nagoya Japan

**Keywords:** immune checkpoint inhibitor, ipilimumab, melanoma, nivolumab

## Abstract

Combination therapy with nivolumab and ipilimumab (NIVO+IPI) is highly effective in treating advanced malignant melanoma (MM) but it is associated with a high incidence of treatment‐related adverse events (TRAEs). This retrospective, cohort study evaluated the efficacy and TRAEs of NIVO+IPI in Japanese patients with unresectable stage III and IV MM, comparing outcomes based on the number of treatment cycles and the IPI dose. We reviewed data from 57 patients with advanced or recurrent MM who received NIVO+IPI at the Shizuoka Cancer Center between August 2015 and July 2024. Patients who received two or fewer NIVO + IPI cycles (NIVO+IPI ≤2 cycles) generally had worse Eastern Cooperative Oncology Group performance status and more advanced stages compared to those who received three or more cycles (NIVO+IPI ≥3 cycles). The analysis revealed that the NIVO+IPI ≥3 cycles group had significantly better overall survival compared to the NIVO+IPI ≤2 cycles group, although receiving three or more cycles was not an independent prognostic factor in multivariate analysis. There was no significant difference in the frequency or severity of TRAEs between the two groups, but the incidence of grade ≥3 TRAEs increased significantly between the first and second cycles of NIVO+IPI. Additionally, reducing the IPI dose from 3 mg/kg to 2 mg/kg appeared to lower the risk of grade ≥3 TRAEs. In conclusion, further research is needed to determine the optimal number of NIVO+IPI cycles for Japanese patients with advanced MM. However, assessing efficacy after the second cycle may help avoid unnecessary NIVO+IPI administration. Reducing the IPI dose to 2 mg/kg may also offer a safer treatment approach for these patients.

## 1 INTRODUCTION

The advent of immune checkpoint inhibitors (ICIs) has greatly improved the prognosis of malignant melanoma (MM), establishing ICIs as the cornerstone of systemic therapy for MM. In particular, the combination of nivolumab and ipilimumab (NIVO+IPI) has been found to be more effective than anti‐ programmed cell death protein 1 (PD‐1) antibody monotherapy or ipilimumab monotherapy among ICIs.^1^ However, while NIVO+IPI has high efficacy, it is also associated with a high incidence of treatment‐related adverse events (TRAEs). A pooled safety analysis of NIVO+IPI in patients with advanced MM showed that 87.5% of patients experienced TRAEs of any grade, with 41.5% having grade 3 or higher TRAEs. The analysis also reported that both the frequency and severity of TRAEs were significantly higher with NIVO+IPI therapy compared to NIVO or IPI monotherapy.^2^

Standard NIVO+IPI therapy involves four cycles of NIVO (1 mg/kg) combined with IPI (3 mg/kg) followed by NIVO monotherapy. The four‐cycle regimen for NIVO+IPI was initially based on previous induction therapy with four cycles of IPI.^3^ However, the need for four cycles of NIVO+IPI remains unestablished. Regarding NIVO+IPI dosing, it has been reported that reducing the IPI dose to 1 mg/kg while increasing the NIVO dose (3 mg/kg) can lower toxicity.^4^ In IPI monotherapy for patients with unresectable stage III or IV MM, the randomized CA184‐169 phase III trial comparing 10 mg/kg IPI (IPI10) with 3 mg/kg IPI (IPI3) reported an increase inefficacy and toxicity proportional to the IPI dose.^5^ The results suggest that the dose of IPI significantly influences the incidence and severity of TRAEs in NIVO + IPI therapy. Therefore, comparing the efficacy and toxicity based on the number of NIVO+IPI cycles and the dose of IPI in patients with MM is highly relevant. In addition, most clinical trials of NIVO+IPI in patients with MM have been conducted in Western populations, with limited data on efficacy and toxicity in Asian populations. To clarify whether NIVO+IPI is necessary for more than three cycles and whether the dose of IPI should be 3 mg/kg in advanced‐stage MM, especially in Asian patients, we compared the efficacy and TRAEs of NIVO+IPI in Japanese patients with unresectable stage III and stage IV MM, focusing on the number of NIVO+IPI cycles and the dose of IPI.

## 2 MATERIALS AND METHODS

### 2.1 Study population and data collection

This retrospective, cohort study was conducted at the Shizuoka Cancer Center in Japan and was approved by the Center's institutional review board. We reviewed the records of patients with advanced or recurrent MM who received NIVO+IPI between August 2015 and July 2024. The study included patients with a histologically confirmed diagnosis of MM who underwent at least one cycle of NIVO+IPI, consisting of NIVO (80 mg/body) and IPI (2–3 mg/kg) administered once every 3 weeks for up to four cycles. Focusing on the dose dependence of IPI efficacy, the IPI dose reduction group was set at 2 mg/kg.

Clinical data collected included patients' age, sex, Eastern Cooperative Oncology Group performance status (ECOG‐PS) score, primary tumor location, history of treatment with surgery and adjuvant therapy, metastatic organs, *BRAF* gene mutation status, TRAEs, and lactate dehydrogenase (LDH) levels at the start of NIVO+IPI therapy. The American Joint Committee on Cancer, 8th Edition, did not include mucosal melanomas in genital, anal, or urinary sites, but evaluated M staging for stage IV based on the cutaneous melanoma criteria. The Common Terminology Criteria for Adverse Events, version 5.0, was used to grade the severity of TRAEs. All personal data were handled in strict accordance with the ethical guidelines stipulated by the 1964 Declaration of Helsinki.

### 2.2 Efficacy assessment

In the main analysis, patients were divided into two groups: those who received NIVO+IPI once or twice (NIVO+IPI ≤2 cycles) and those who received NIVO+IPI three or four times (NIVO+IPI ≥3 cycles). The primary outcomes were objective response rate (ORR), progression‐free survival (PFS), overall survival (OS), and frequency of TRAEs. As a sub‐analysis, PFS, OS, and frequency of TRAEs were compared between the 2 mg/kg and 3 mg/kg IPI groups. Treatment response was evaluated using Response Evaluation Criteria in Solid Tumors, version 1.1. The ORR was defined as the proportion of patients who achieved a complete response (CR) or partial response (PR).

### 2.3 Statistical analysis

Baseline characteristics were compared using the Mann–Whitney *U* test for continuous variables and the chi‐squared test and Fisher's exact test for categorical variables. The chi‐squared and Fisher's exact tests were also used to analyze the ORR and frequency of TRAEs. OS and PFS were estimated using the Kaplan–Meier method. Differences in OS and PFS across treatments were assessed using log‐rank tests. Cox regression analysis was performed to calculate hazard ratios (HRs) for covariates affecting OS and PFS. *p*‐values of <0.05 were considered statistically significant. All analyses were performed using EZR version 1.55 for Windows XP‐11.

## 3 RESULTS

### 3.1 Baseline patient characteristics

The baseline characteristics of the patients are summarized in Table 1. A total of 57 patients with MM were enrolled. Of these, 28 patients (49.1%) received one or two cycles of NIVO+IPI, while 29 patients (50.9%) received three or four cycles. The median follow‐up period was longer in the NIVO+IPI ≥3 cycles group than in the NIVO+IPI ≤2 cycles group (19.1 vs 11.1 months). Regarding age at NIVO+IPI initiation, the NIVO+IPI ≥3 cycles group tended to be younger than the NIVO+IPI ≤2 cycles group (median age, 58 vs 63 years, *p* = 0.174). Approximately half of the patients in both groups were female (NIVO+IPI ≤2; *n* = 14, 50%, NIVO+IPI ≥3; *n* = 14, 48.3%). Overall, most patients had an ECOG‐PS of 0 or 1 (*n* = 52, 91.2%), with a trend toward better PS in the NIVO+IPI ≥3 cycles group (100% vs 82.1%, *p* = 0.082). No significant difference was observed in the primary tumor site between the two groups, with cutaneous (*n* = 19, 33.3%), acral (*n* = 9, 15.8%), mucosal (*n* = 20, 35.1%), uveal (*n* = 7, 12.3%), and unknown primary (*n* = 2, 3.5%) tumors overall. In terms of stage, five patients (8.8%) were in stage III, six (10.5%) were in stage IV (M1b), 42 (73.7%) were in stage IV (M1c), and four (7.0%) were in stage IV (M1d). Patients in the NIVO+IPI ≤2 cycles group tended to be in significantly worse stages (*p* = 0.003). More than half of the patients had normal LDH levels (59.7%) and no *BRAF* gene mutation (64.9%). Many patients (*n* = 40, 70.2%) did not undergo surgical treatment for their primary tumor, and many patients (*n* = 37, 65.0%) did not received adjuvant therapy. Nine patients (15.8%) received NIVO+IPI once, 19 patients (33.3%) twice, 10 patients (17.5%) three times, and 19 patients (33.3%) four times. Regarding the dose of IPI, 48 patients (84.2%) were treated with 2 mg/kg, and nine patients (15.8%) with 3 mg/kg. There were no significant differences in baseline characteristics of patients with MM classified by IPI dose between the 2 mg/kg and 3 mg/kg groups (Table 2).

**TABLE 1:** Baseline characteristics of patients with MM.

Characteristic	Patient groups			*p‐*value*
	Total	NIVO+IPI≦2 cycles	NIVO+IPI≧3 cycles	
Patients	57 (100)	28 (49.1)	29 (50.9)	
Age (years)				0.174
Median [range]	61 [42– 84]	63 [44–84)	58 [42–74]	
Sex				1.000
Male	29 (50.9)	14 (50.0)	15 (51.7)	
Female	28 (49.1)	14 (50.0)	14 (48.3)	
ECOG‐PS score				0.082
0	42 (73.7)	17 (60.7)	25 (86.2)	
1	10 (17.5)	6 (21.4)	4 (13.8)	
2	2 (3.5)	2 (7.1)	0 (0.0)	
3	2 (3.5)	2 (7.1)	0 (0.0)	
4	1 (1.8)	1 (3.6)	0 (0.0)	
Primary site				0.865
Cutaneous (non‐acral)	19 (33.3)	11 (39.3)	8 (27.6)	
Acral	9 (15.8)	4 (14.3)	5 (17.2)	
Mucosal	20 (35.1)	8 (28.6)	12 (41.4)	
Uveal	7 (12.3)	4 (14.3)	3 (10.3)	
Unknown primary	2 (3.5)	1 (3.6)	1 (3.6)	
Stage				0.003
III	5 (8.8)	0 (0.0)	5 (17.2)	
IV (M1a)	0 (0.0)	0 (0.0)	0 (0.0)	
IV (M1b)	6 (10.5)	0 (0.0)	6 (20.7)	
IV (M1c)	42 (73.7)	25 (89.3)	17 (58.6)	
IV (M1d)	4 (7.0)	3 (10.7)	1 (3.4)	
LDH value				0.106
<ULN	23 (40.4)	8 (28.6)	15 (51.7)	
≥ULN	34 (59.7)	20 (71.4)	14 (48.3)	
BRAF				0.433
Mutant	11 (19.3)	6 (21.4)	5 (17.2)	
Wild type	37 (64.9)	16 (57.1)	21 (72.4)	
Not investigated	9 (15.8)	6 (21.4)	3 (10.3)	
Surgery for primary site				0.395
Yes	17 (29.8)	10 (35.7)	7 (24.1)	
No	40 (70.2)	18 (64.3)	22 (75.9)	
Adjuvant therapy				0.386
None	37 (65.0)	17 (60.7)	20 (69.0)	
Nivolumab or pembrolizumab	14 (24.6)	6 (21.4)	8 (27.6)	
BRAF/MEKi	2 (3.5)	2 (7.1)	0 (0.0)	
Others	4 (7.0)	3 (10.7)	1 (3.4)	
ICI treatment prior to NIVO + IPI				0.429
Yes	26 (45.7)	11 (39.3)	15 (51.7)	
No	31 (54.4)	17 (60.7)	14 (48.3)	
BRAF/MEKi prior to NIVO + IPI				0.253
Yes	7 (12.3)	5 (17.9)	2 (6.9)	
No	50 (57.7)	23 (82.1)	27 (93.1)	
Number of NIVO+IPI treatments				
1	9 (15.8)	9 (32.1)	–	
2	19 (33.3)	19 (67.9)	–	
3	10 (17.5)	–	10 (34.5)	
4	19 (33.3)	–	19 (65.5)	
Dose of ipilimumab				0.297
2 mg/kg	48 (84.2)	22 (78.6)	26 (89.7)	
3 mg/kg	9 (15.8)	6 (21.4)	3 (10.3)	
Reasons for the termination of NIVO + IPI				<0.001
Complete	19 (33.3)	0 (0.0)	19 (65.5)	
Progressive disease	6 (10.5)	6 (21.4)	0 (0.0)	
TRAE	21 (36.8)	12 (42.9)	9 (31.0)	
Worsening of the condition of a patient	9 (15.8)	8 (28.6)	1 (3.4)	
Patient selection	2 (3.5)	2 (7.1)	0 (0.0)	
Outcome				0.530
Dead	44 (77.2)	23 (82.1)	21 (72.4)	
Alive	13 (22.8)	5 (17.9)	8 (27.6)	

*Note:* Data are presented as *n* (%) unless otherwise specified.

Abbreviations: BRAF/MEKi, BRAF/MEK inhibitor; ECOG‐PS, Eastern Cooperative Oncology Group Performance Status; ICI, immune check‐point inhibitor; LDH, lactate dehydrogenase; MM, malignant melanoma; NIVO+IPI, nivolumab plus ipilimumab therapy; TRAE, treatment‐related adverse event; ULN, upper limit of normal.

* Statistically significant: *p* < 0.05.

**TABLE 2:** Baseline characteristics of patients with MM grouped by ipilimumab dose.

Characteristic	Patient group (%)			*p‐*value*
	Total	IPI = 2 mg/kg	IPI = 3 mg/kg	
Patients	57 (100)	48 (84.2)	9 (15.8)	
Age (years)				0.609
Median [range]	61 [42– 84]	62 [42–84]	60 [52–70]	
Sex				0.470
Male	29 (50.9)	23 (47.9)	6 (66.7)	
Female	28 (49.1)	25 (52.1)	3 (33.3)	
ECOG‐PS score				0.664
0	42 (73.7)	35 (72.9)	7 (77.8)	
1	10 (17.5)	9 (18.8)	1 (11.1)	
2	2 (3.5)	2 (4.2)	0 (0.0)	
3	2 (3.5)	1 (2.1)	1 (2.1)	
4	1 (1.8)	1 (2.1)	0 (0.0)	
Primary site				0.967
Cutaneous (non‐acral)	19 (33.3)	15 (31.2)	4 (44.4)	
Acral	9 (15.8)	8 (16.7)	1 (11.1)	
Mucosal	20 (35.1)	17 (35.4)	3 (33.3)	
Uveal	7 (12.3)	6 (12.5)	1 (11.1)	
Unknown primary	2 (3.5)	2 (4.2)	0 (0.0)	
Stage				0.511
III	5 (8.8)	5 (10.4)	0 (0.0)	
IV (M1a)	0 (0.0)	0 (0.0)	0 (0.0)	
IV (M1b)	6 (10.5)	6 (12.5)	0 (0.0)	
IV (M1c)	42 (73.7)	33 (68.8)	9 (100.0)	
IV (M1d)	4 (7.0)	4 (8.3)	0 (0.0)	
LDH value				1.000
<ULN	23 (40.4)	19 (39.6)	4 (44.4)	
≥ULN	34 (59.7)	29 (60.4)	5 (55.6)	
BRAF				0.334
Mutant	11 (19.3)	8 (16.7)	3 (33.3)	
Wild type	37 (64.9)	33 (68.8)	4 (44.4)	
Not investigated	9 (15.8)	7 (14.6)	2 (22.2)	
Number of NIVO+IPI treatments				0.726
1	9 (15.8)	7 (14.6)	2 (22.2)	
2	19 (33.3)	15 (31.2)	4 (44.4)	
3	10 (17.5)	9 (18.8)	1 (11.1)	
4	19 (33.3)	17 (35.4)	2 (22.2)	
Outcome				0.101
Dead	44 (77.2)	35 (72.9)	9 (100.0)	
Alive	13 (22.8)	13 (27.1)	0 (0.0)	

*Note:* Data are presented as *n* (%) unless otherwise specified.

Abbreviations: ECOG‐PS, Eastern Cooperative Oncology Group Performance Status; LDH, lactate dehydrogenase; MM, malignant melanoma; NIVO+IPI, nivolumab plus ipilimumab therapy; TRAE, treatment‐related adverse event; ULN, upper limit of normal.

* Statistically significant: *p* < 0.05.

### 3.2 Objective response

In this cohort, the overall ORR was 19.3% (5.3% CR, 14.0% PR) (Table 3). The ORRs were not significantly different, but the NIVO+IPI ≤2 cycles group showed a trend toward a higher ORR than the NIVO+IPI ≥3 cycles group (25.0% vs 13.8%, *p* = 0.331) (Table 3). Progressive disease was the most common response in both the NIVO+IPI ≤2 cycles and NIVO+IPI ≥3 cycles groups (42.9% vs 65.5%).

**TABLE 3:** Overall response between the NIVO+IPI ≤2 cycles and NIVO+IPI ≥3 cycles groups in patients with MM.

	Patient group			*p‐*value*
	Total *n* = 57	NIVO+IPI≦2 cycles *n* = 28	NIVO+IPI≧3 cycles *n* = 29	
Best overall response				0.043
Complete response	3 (5.3)	1 (3.6)	2 (6.9)	
Partial response	8 (14.0)	6 (21.4)	2 (6.9)	
Stable disease	10 (17.5)	4 (14.3)	6 (20.7)	
Progressive disease	31 (54.4)	12 (42.9)	19 (65.5)	
Not evaluable	5 (8.8)	5 (17.9)	0 (0.0)	
ORR, %	19.3	25.0	13.8	0.331

*Note:* Data are presented as *n* (%) unless otherwise specified.

Abbreviations: MM, malignant melanoma; NIVO+IPI, nivolumab plus ipilimumab therapy; ORR, objective response rate.

* Statistically significant: *p* < 0.05.

In the NIVO+IPI ≥3 cycles group, six patients were evaluated for objective response after two cycles. Two patients had stable disease, and four patients had progressive disease after two cycles. However, all six patients showed progressive disease after four cycles.

The sub‐analysis compared the ORR between the IPI 2 mg/kg and IPI 3 mg/kg groups. The ORRs were 16.7% in the IPI 2 mg/kg group and 33.3% in the IPI 3 mg/kg group (*p* = 0.354) (Table 4).

**TABLE 4:** Overall response between the IPI 2 mg/kg and IPI 3 mg/kg groups in patients with MM.

	Patient group			*p‐*value*
	Total *n* = 57	Ipilimumab 2 mg/kg *n* = 48	Ipilimumab 3 mg/kg *n* = 9	
Best overall response				0.428
Complete response	3 (5.3)	3 (6.2)	0 (0.0)	
Partial response	8 (14.0)	5 (10.4)	3 (33.3)	
Stable disease	10 (17.5)	9 (18.8)	1 (11.1)	
Progressive disease	31 (54.4)	27 (56.2)	4 (44.4)	
Not evaluable	5 (8.8)	4 (8.3)	1 (11.1)	
ORR, %	21.6	16.7	33.3	0.354

*Note:* Data are presented as *n* (%) unless otherwise specified.

Abbreviations: IPI, ipilimumab; MM, malignant melanoma; ORR, objective response rate.

* Statistically significant: *p* < 0.05.

### 3.3 PFS and OS

**FIGURE 1:**
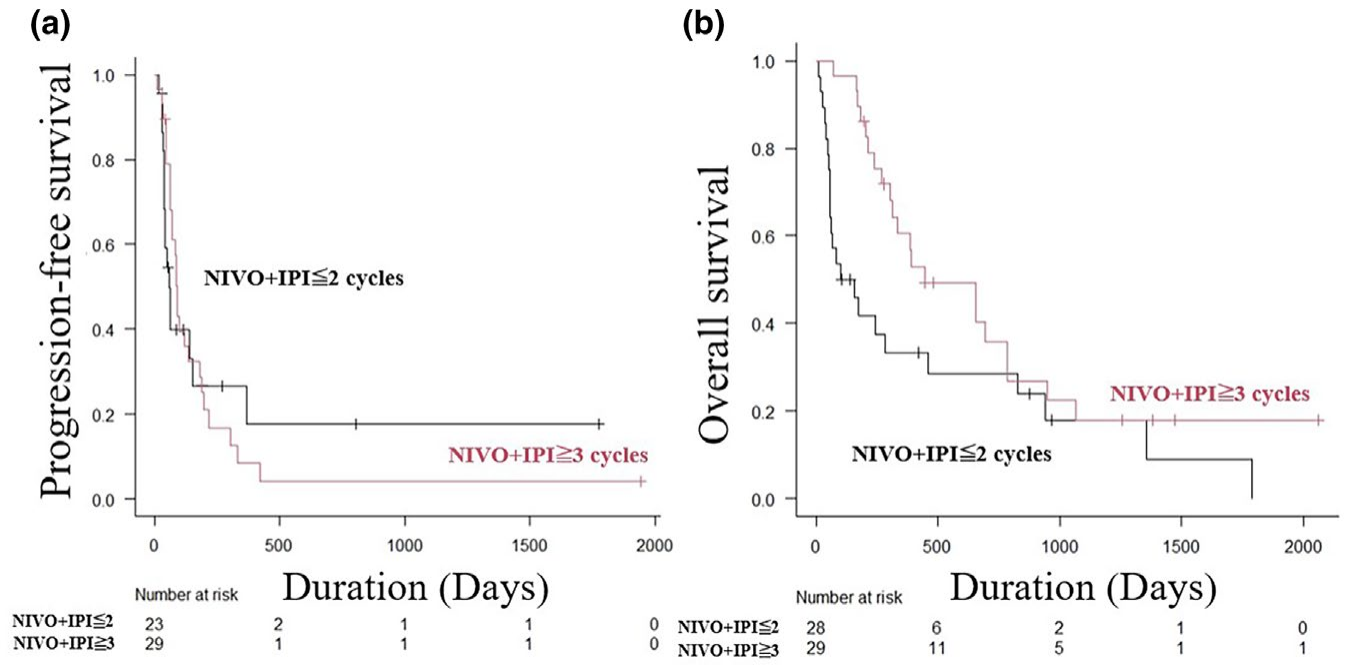
Kaplan–Meier analysis of progression‐free survival (PFS) and overall survival (OS) between the nivolumab plus ipilimumab therapy (NIVO + IPI) ≤2 cycles and NIVO + IPI ≥3 cycles groups. (a) Kaplan–Meier analysis of PFS in the NIVO+IPI ≤2 cycles and NIVO+IPI ≥3 cycles groups. The difference in PFS between the NIVO+IPI ≤2 cycles and NIVO+IPI ≥3 cycles groups was not statistically significant (median PFS time, 2.0 months vs 3.0 months, log‐rank test, *p* = 0.944). (b) Kaplan–Meier analysis of OS in the NIVO+IPI ≤2 cycles and NIVO+IPI ≥3 cycles groups. The NIVO+IPI ≥3 cycles group had significantly better OS than the NIVO+IPI ≤2 cycles group (median OS, 14.7 months vs 4.2 months, log‐rank test, *p* = 0.034).

The overall median PFS was 2.3 months. There were no statistically significant differences in PFS between the NIVO+IPI ≤2 cycles and NIVO+IPI ≥3 cycles groups (median PFS, 2.0 months vs 3.0 months, log‐rank test, *p* = 0.944) (Figure 1a). Regarding OS, the overall median OS was 9.2 months, with the NIVO+IPI ≥3 cycles group showing significantly better OS than the NIVO+IPI ≤2 cycles group (median OS, 14.7 months vs 4.2 months, log‐rank test, *p* = 0.034) (Figure 1b). Specifically, the 1‐year OS was 33.3% vs 60.5% (HR, 0.340; 95% CI 0.160–0.725; *p* = 0.005) and the 2‐year OS was 28.6% vs 35.7% (HR, 0.501; 95% CI 0259–0.970; *p* = 0.004) in the NIVO+IPI ≤2 cycles and NIVO+IPI ≥3 cycles, respectively.

**FIGURE 2:**
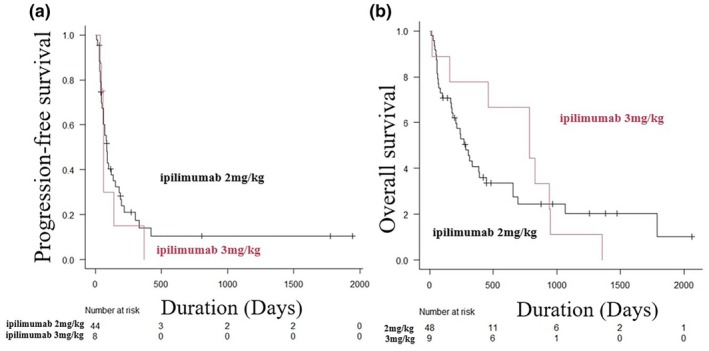
Kaplan–Meier analysis of progression‐free survival (PFS) and overall survival (OS) between the ipilimumab (IPI) 2 mg/kg and IPI 3 mg/kg groups. (a) Kaplan–Meier analysis of PFS in the IPI 2 mg/kg and IPI 3 mg/kg groups. The difference in PFS between the IPI 2 mg/kg and IPI 3 mg/kg groups was not statistically significant (median PFS time, 2.9 months vs 2.1 months, log‐rank test, *p* = 0.479). (b) Kaplan–Meier analysis of OS in the IPI 2 mg/kg and IPI 3 mg/kg groups. The difference in OS between the IPI 2 mg/kg and IPI 3 mg/kg groups was not statistically significant (median OS time, 9.3 months vs 25.4 months, log‐rank test, *p* = 0.479).

The sub‐analysis evaluated PFS and OS in the IPI 2 mg/kg (48 patients) and IPI 3 mg/kg (nine patients) groups. There was no significant difference in PFS between the IPI 2 mg/kg and IPI 3 mg/kg groups (median PFS time, 2.9 months vs 2.1 months, log‐rank test, *p* = 0.479) (Figure 2a). There was also no significant difference in OS between the two groups (median OS time, 9.3 months vs 25.4 months, log‐rank test, *p* = 0.479) (Figure 2b).

### 3.4 Univariate and multivariate analysis of potential prognostic factors for PFS and OS

In the univariate analysis, only age (HR, 1.040, 95% CI 1.004–1.078, *p* = 0.030) was significantly associated with PFS (Table 5). For OS, the number of NIVO+IPI cycles (HR, 0.531, 95% CI 0.292–0.965, *p* = 0.038), age (HR, 1.065, 95% CI 1.027–1.105, *p* < 0.001), ECOG‐PS (HR, 52.27, 95% CI 9.73–280.7, *p* < 0.001), stage (M1d, HR, 6.809, 95% CI 1.100–42.17, *p* = 0.039), LDH value (HR, 2.099, 95% CI 1.109–3.973, *p* = 0.023), and presence of TRAEs ≥ grade 3 (HR, 0.311, 95% CI 0.162–0.596, *p* < 0.001) were significantly associated with OS (Table 6). In the multivariate analysis, age (HR, 1.079, 95% CI 1.030–1.130, *p* = 0.001) and primary site (cutaneous, HR, 3.708, 95% CI 1.126–12.22, *p* = 0.031) were identified as independent prognostic factors for PFS (Table 5). For OS, the independent prognostic factors were age (HR, 1.092, 95% CI 1.042–1.144, *p* < 0.001), ECOG‐PS (HR, 574.8, 95% CI 29.87–11 060, *p* < 0.001), primary site (cutaneous, HR, 3.029, 95% CI1.012–9.070, *p* = 0.048, unknown primary, HR, 0.034, 95% CI 0.002–0.544, *p* = 0.017), and the presence of TRAEs ≥ grade 3 (HR, 0.214, 95% CI 0.093–0.496, *p* < 0.001) (Table 6). The number of NIVO+IPI cycles was not an independent prognostic factor in PFS and OS.

**TABLE 5:** Univariate and multivariate analysis of potential prognostic factors for PFS.

	Univariate analysis			Multivariate analysis		
	Hazard ratio	95% CI	*p‐*value*	Hazard ratio	95% CI	*p‐*value*
NIVO+IPI≦2 cycles	Reference			Reference		
NIVO+IPI≧3 cycles	0.978	0.522–1.833	0.944	1.274	0.581–2.796	0.546
Age	1.040	1.004–1.078	**0.030**	1.079	1.030–1.130	**0.001**
ECOG‐PS score 0–1	Reference			Reference		
ECOG‐PS score ≥2	6.253	0.744–52.57	0.092	5.927	0.380–92.40	0.204
Primary site						
Acral	Reference			Reference		
Cutaneous (non‐acral)	2.755	0.983–7.727	0.054	3.708	1.126–12.22	**0.031**
Mucosal	1.792	0.681–4.713	0.237	1.818	0.603–5.479	0.288
Uveal	0.987	0.269–3.628	0.984	0.743	0.184–2.990	0.675
Unknown primary	0.598	0.072–4.992	0.635	0.179	0.014–2.323	0.188
Stage						
III	Reference			Reference		
M1b	0.667	0.155–2.867	0.587	0.396	0.075–2.101	0.277
M1c	1.189	0.360–3.928	0.777	0.629	0.143–2.763	0.534
M1d	3.449	0.670–17.74	0.139	1.059	0.140–8.011	0.956
LDH value <ULN	Reference			Reference		
LDH value ≥ULN	1.976	1.055–3.699	0.033	1.789	0.757–4.229	0.185
Ipilimumab = 2 mg/kg	Reference			Reference		
Ipilimumab = 3 mg/kg	1.341	0.591–3.045	0.483	2.202	0.766–6.330	0.143
BRAF wild type	Reference			NI		
BRAF mutant	1.187	0.514–2.744	0.688			
Adjuvant therapy				NI		
None	Reference					
Nivolumab or pembrolizumab	1.929	0.978–3.805	0.058			
BRAF/MEKi	3.079	0.401–23.660	0.280			
Others	0.939	0.282–3.123	0.918			
Grade≧3 TRAE						
None	Reference			Reference		
Yes	0.676	0.365–1.253	0.214	0.772	0.343–1.737	0.532

*Note:* *p*‐value of 0.05 or less are bolded.

Abbreviations: BRAF/MEKi, BRAF/MEK inhibitor; CI, confidence interval; ECOG‐PS, Eastern Cooperative Oncology Group Performance Status; LDH, lactate dehydrogenase; MM, malignant melanoma; NI, not included; NIVO+IPI, nivolumab plus ipilimumab therapy; PFS, progression free survival; TRAE, treatment‐related adverse event; ULN, upper limit of normal.

* Statistically significant: *p* < 0.05.

**TABLE 6:** Univariate and multivariate analysis of potential prognostic factors for OS.

	Univariate analysis			Multivariate analysis		
	Hazard ratio	95% CI	*p‐*value*	Hazard ratio	95% CI	*p‐*value*
NIVO+IPI≦2 cycles	Reference			Reference		
NIVO+IPI≧3 cycles	0.531	0.292–0.965	0.038	0.756	0.344–1.664	0.487
Age	1.065	1.027–1.105	<0.001	1.092	1.042–1.144	<0.001
ECOG‐PS score 0–1	Reference			Reference		
ECOG‐PS score≧2	52.27	9.73–280.7	<0.001	574.8	29.87–11 060	<0.001
Primary site						
Acral	Reference			Reference		
Cutaneous	1.921	0.765–4.825	0.165	3.029	1.012–9.070	0.048
Mucosal	1.085	0.443–2.657	0.859	2.102	0.768–5.750	0.148
Uveal	1.258	0.394–4.020	0.699	1.097	0.314–3.839	0.885
Unknown primary	0.589	0.071–4.893	0.624	0.034	0.002–0.544	0.017
Stage						
III	Reference			Reference		
M1b	1.191	0.215–6.600	0.841	0.942	0.137–6.470	0.951
M1c	2.410	0.578–10.05	0.227	1.947	0.358–10.58	0.441
M1d	6.809	1.100–42.17	0.039	0.273	0.016–4.607	0.368
LDH value <ULN	Reference			Reference		
LDH value ≥ULN	2.099	1.109–3.973	0.023	2.521	0.994–6.394	0.051
Ipilimumab = 2 mg/kg	Reference			Reference		
Ipilimumab = 3 mg/kg	0.865	0.409–1.828	0.704	0.633	0.241–1.662	0.353
BRAF wild type	Reference			NI		
BRAF mutant	0.989	0.445–2.198	0.979			
Adjuvant therapy				NI		
None	Reference					
Nivolumab or pembrolizumab	1.305	0.662–2.576	0.442			
BRAF/MEKi	1.379	0.326–5.839	0.663			
Others	0.275	0.037–2.025	0.205			
Grade≧3 TRAE						
None	Reference			Reference		
Yes	0.311	0.162–0.596	<0.001	0.214	0.093–0.496	<0.001

Abbreviations: BRAF/MEKi, BRAF/MEK inhibitor; CI, confidence interval; ECOG‐PS, Eastern Cooperative Oncology Group Performance Status; LDH, lactate dehydrogenase; MM, malignant melanoma; NI, not included; NIVO+IPI, nivolumab plus ipilimumab therapy; OS, overall survival; TRAE, treatment‐related adverse event; ULN, upper limit of normal.

* Statistically significant: *p* < 0.05.

### 3.5 Toxicity

As for adverse events presumed to be related to NIVO+IPI, 52 patients (91.2%) experienced any grade TRAEs, and 25 patients (43.9%) had grade ≥3 TRAEs (Table 7). The NIVO+IPI ≥3 cycles group tended to have more rash (*p* = 0.061), itching (*p* = 0.014), and cough (*p* = 0.023) than the NIVO+IPI ≤2 cycles group. In both groups, 30%–40% of patients had fever and elevated aspartate aminotransferase (AST) and alanine aminotransferase (ALT) levels. In the grade ≥3 TRAE subgroup, both the NIVO+IPI ≤2 cycles and NIVO+IPI ≥3 cycles groups had similar rates of elevated AST/ALT (*n* = 7, 25% vs *n* = 5, 17.2%, *p* = 0.530), fever (*n* = 3, 10.7% vs *n* = 5, 17.2%, *p* = 0.706), and colitis (*n* = 2, 7.1% vs *n* = 2, 6.9%, *p* = 1.000) (Table 7). In the IPI 2 mg/kg and IPI 3 mg/kg groups, 91.7% and 88.9% of patients respectively, had any‐grade TRAEs (*p* = 1.000), while 37.5% and 77.8% respectively had grade ≥3 TRAEs (*p* = 0.034) (Table 8).

**TABLE 7:** Treatment‐related adverse events by NIVO+IPI treatment frequency group.

Event	Total	NIVO+IPI≦2 cycles *n* = 28	NIVO+IPI≧3 cycles *n* = 29	*p‐*value*
	No. of events (%)			
Any grade of TRAE	52 (91.2)	24 (85.7)	28 (96.6)	0.194
Rash	24 (42.1)	8 (28.6)	16 (55.2)	0.061
Itchy	22 (38.6)	6 (21.4)	16 (55.2)	**0.014**
Fever	21 (36.8)	11 (39.3)	10 (34.5)	0.787
Increased AST/ALT level	21 (36.8)	11 (39.3)	10 (34.5)	0.787
Fatigue	16 (28.1)	8 (28.6)	8 (27.6)	1.000
Diarrhea/Colitis	10 (17.5)	3 (10.7)	7 (24.1)	0.297
Coughing	6 (10.5)	0 (0.0)	6 (20.7)	0.023
Pneumonitis	5 (8.8)	2 (7.1)	3 (10.3)	1.000
Hypophysitis	5 (8.8)	3 (10.7)	2 (6.9)	0.670
Hyperthyroidism	3 (5.3)	0 (0.0)	3 (10.3)	0.237
Oral mucositis	3 (5.3)	1 (3.6)	2 (6.9)	1.000
Acute kidney injury	2 (3.5)	1 (3.6)	1 (3.4)	1.000
Adrenal insufficiency	2 (3.5)	0 (0.0)	2 (6.9)	0.491
Increased serum amylase level	1 (1.8)	1 (3.6)	0 (0.0)	1.000
Dysgeusia	1 (1.8)	1 (3.6)	0 (0.0)	1.000
Type I diabetes mellitus	1 (1.8)	1 (3.6)	0 (0.0)	1.000
Thromboembolism	1 (1.8)	0 (0.0)	1 (3.4)	1.000
Increased creatine Kinase	1 (1.8)	0 (0.0)	1 (3.4)	1.000
Depilation	1 (1.8)	0 (0.0)	1 (3.4)	1.000
Gastric ulcer	1 (1.8)	0 (0.0)	1 (3.4)	1.000
Nausea	1 (1.8)	0 (0.0)	1 (3.4)	1.000
Grade≧3 TRAE	25 (43.9)	12 (42.9)	13 (44.8)	1.000
Increased AST/ALT level	12 (21.1)	7 (25.0)	5 (17.2)	0.530
Fever	8 (14.0)	3 (10.7)	5 (17.2)	0.706
Diarrhea/Colitis	4 (7.0)	2 (7.1)	2 (6.9)	1.000
Pneumonitis	2 (3.5)	1 (3.6)	1 (3.4)	1.000
Rash	2 (3.5)	0 (0.0)	2 (6.9)	1.000
Hyperthyroidism	1 (1.8)	0 (0.0)	1 (3.4)	1.000
Type I diabetes mellitus	1 (1.8)	1 (3.6)	0 (0.0)	1.000
Thromboembolism	1 (1.8)	0 (0.0)	1 (3.4)	1.000

*Note:* Data are presented as *n* (%) unless otherwise specified. *p*‐values of 0.05 or less are bolded.

Abbreviations: ALT, alanine aminotransferase; AST, aspartate aminotransferase; NIVO+IPI, nivolumab plus ipilimumab therapy; TRAE, treatment‐related adverse event.

* Statistically significant: *p* < 0.05.

**TABLE 8:** Treatment‐related adverse events by IPI dose group.

Event	Total *n* = 57	IPI 2 mg/kg *n* = 48	IPI 3 mg/kg *n* = 9	*p‐*value*
	No. of events			
Any grade of TRAE				1.000
Yes	52 (91.2)	44 (91.7)	8 (88.9)	
No	5 (8.8)	4 (8.3)	1 (11.1)	
Grade≧3 TRAE				0.034
Yes	25 (43.9)	18 (37.5)	7 (77.8)	
No	32 (56.1)	30 (62.5)	2 (22.2)	

*Note:* Data are presented as *n* (%).

Abbreviations: IPI, ipilimumab; TRAE, treatment‐related adverse event.

* Statistically significant: *p* < 0.05.

Additionally, the timing of the onset for grade ≥3 TRAEs was examined (Table 9). The incidence of grade ≥3 TRAEs was 3.5% from the first to the second administration of NIVO+IPI. From the second dose to the third dose, the incidence increased to 20.8%; from the third dose to the fourth dose, it increased to 27.6%; and after the fourth dose, it reached 26.3%. A significant increase in the incidence of grade ≥3 TRAEs was observed from the first to the second dose (first vs second, *p* = 0.005), but no significant increase occurred after the second dose (second vs third, *p* = 0.498, third vs fourth, *p* = 0.923).

**TABLE 9:** Relationship between the number of NIVO+IPI cycles and the occurrence of treatment‐related grade≧3 adverse events.

Patients with received NIVO+IPI at least	Once	Twice	3 times	4 times
Patients, *n* (%)	57 (100)	48 (84.2)	29 (50.9)	19 (33.3)
Number of patients with grade≧3 adverse events until the next dose	2	10	8	5
Incidence of grade≧3 adverse events until the next dose, %	3.5	20.8 (vs once, *p* = 0.005*)	27.6 (vs twice, *p* = 0.498)	26.3 (vs 3 times, *p* = 0.923)

Abbreviation: NIVO+IPI, nivolumab plus ipilimumab therapy.

* Statistically significant: *p* < 0.05.

## 4 DISCUSSION

This study compared the efficacy and toxicity of NIVO+IPI based on administration frequency in patients with advanced‐stage MM. The analysis revealed that the NIVO+IPI ≥3 cycles group had significantly better OS than the NIVO+IPI ≤2 cycles group; however, NIVO+IPI ≥3 cycles was not a favorable prognostic factor in the multivariate analysis. Moreover, no significant difference was found in the frequency and severity of TRAEs between the two groups. In addition, the incidence of grade ≥3 TRAEs significantly increased between the first and second cycles of NIVO+IPI. Finally, reducing the IPI dose from 3 mg/kg to 2 mg/kg may reduce the risk of grade ≥3 TRAEs in NIVO+IPI therapy.

The main aim of this study was to compare the efficacy of NIVO+IPI in patients with unresectable stage III or stage IV MM between NIVO+IPI ≤2 cycles and NIVO+IPI ≥3 cycles groups. Interestingly, the NIVO+IPI ≤2 cycles group showed a higher response rate than the NIVO+IPI ≥3 cycles group, although the difference was not statistically significant (25.0% vs 13.8%, *p* = 0.331). This result suggests that NIVO+IPI therapy may show early efficacy, and patients who respond to the therapy may show a good tumor response after two cycles. In fact, the study evaluated an ORR after two cycles in six patients who received NIVO+IPI for three or four cycles. However, no patient showed an improved ORR after the third or fourth cycle. Maeda et al. reported that, in Japanese patients with stage IV MM treated with NIVO+IPI, none of the patients who showed progressive disease after two cycles of therapy experienced improved tumor response with subsequent NIVO+IPI treatment.^6^ Similarly, Postow et al. conducted a prospective trial in patients with unresectable stage III or stage IV MM, intentionally completing two cycles of NIVO+IPI.^7^ In this study, patients with no new lesions or tumor growth >4% after two cycles of NIVO+IPI were transitioned to NIVO monotherapy, while those with new metastases or tumor growth >4% continued NIVO+IPI therapy. Of the 19 patients who continued NIVO+IPI, only two (11%) patients ultimately responded. They concluded that the efficacy of the standard 4‐dose NIVO + IPI induction therapy in MM was likely dependent on the first two cycles. These findings suggest that there may be little benefit in continuing NIVO+IPI beyond two cycles if the response is poor.

In this study, the NIVO+IPI ≥3 cycles group had significantly better OS than the NIVO+IPI ≤2 cycles group (Figure 1b). However, the NIVO+IPI ≥3 cycles group tended to have better ECOG‐PS and stage, which may have improved OS. In the univariate analysis, NIVO+IPI ≥3 cycles was a favorable prognostic factor (HR, 0.531, 95% CI 0.292–0.965, *p* = 0.038); however, it was not an independent factor in the multivariate analysis (HR, 0.756, 95% CI 0.344–1.664, *p* = 0.487). The poorer prognosis in the NIVO+IPI ≤2 cycles group during the first few years may be partly due to 28.6% of the patients who were unable to continue treatment after two cycles due to a deterioration in their condition (Figure 1b). Similarly, Fujisawa et al. reported no significant difference in OS between the NIVO+IPI ≤2 cycles and NIVO+IPI ≥3 cycles groups in a cohort of 97 Japanese patients with advanced MM.^8^ However, since most patients in the NIVO+IPI ≤2 cycles group discontinued therapy after two cycles due to disease progression, tumor growth, or TRAEs, it cannot be definitively concluded that NIVO+IPI should be intentionally completed in two cycles. Prospective trials evaluating elective NIVO+IPI treatment in two cycles in Asian populations are needed.

There are numerous reports^9^, ^10^, ^11^ indicating that the occurrence of TRAEs in ICI treatments for malignant tumors is a favorable prognostic factor, with grade ≥3 TRAEs identified as a favorable prognostic factor in the present multivariate analysis (Table 6). No significant differences in toxicity were observed between the NIVO+IPI ≤2 cycles and NIVO+IPI ≥3 cycles groups for any‐grade and grade ≥3 TRAEs. The observed trend of an increased incidence of grade ≥3 TRAEs after the second administration of NIVO+IPI highlights a critical period where adverse events may peak. This significant rise between the first and second doses (*p* = 0.005) suggests that early immune activation may play a pivotal role in triggering severe adverse effects. However, the lack of significant changes in incidence between subsequent cycles indicates that patients experiencing severe TRAEs are likely to do so early in treatment.

The present study also compared the efficacy and toxicity of different doses of IPI in NIVO+IPI therapy. No significant differences in ORR, PFS, and OS were found between the IPI 2 mg/kg and 3 mg/kg groups; however, the 2 mg/kg group tended to have a worse prognosis than the 3 mg/kg group in OS (median OS time, 9.3 months vs 25.4 months, log‐rank test, *p* = 0.479). This is probably because the effect of IPI is dose‐dependent and there were fewer patients in the 3 mg/kg IPI group. Takahashi et al. reported the efficacy of NIVO+IPI therapy (IPI 3 mg/kg) in 100 Japanese patients with advanced‐stage MM.^12^ They reported an ORR of 24%, a disease control rate of 47%, a median PFS of 3.5 months (95% CI 2.2–4.3), and a median OS of 14.5 months (95% CI 9.2–19.8). Although not directly comparable, the IPI 2 mg/kg group in this study had an ORR of 16.7%, median PFS of 2.9 months, and median OS of 9.3 months, with a slightly better prognosis in the IPI 3 mg/kg group in the previous study. In terms of toxicity, there was no difference in any‐grade TRAEs (91.7% vs 88.9%, *p* = 1.000), but grade ≥3 TRAEs were significantly higher with IPI 3 mg/kg (37.5% vs 77.8, *p* = 0.034). Takahashi et al. also reported that the incidence of any‐grade TRAEs was 89%, and grade ≥3 TRAEs was 56% for NIVO+IPI (IPI 3 mg/kg) in Japanese patients with advanced‐stage MM.^12^ The CheckMate 067 trial evaluated the efficacy and toxicity of NIVO+IPI therapy (IPI 3 mg/kg) in 313 patients with untreated, unresectable stage III or IV MM and reported that grade ≥3 NIVO+IPI‐related TRAEs occurred in 59% of patients.^13^ The CheckMate 511 study compared the efficacy and side effects of NIVO 1 mg/kg plus IPI 3 mg/kg (NIVO1 + IPI3) and NIVO 3 mg/kg plus IPI 1 mg/kg (NIVO3 + IPI1) in 360 patients with unresectable stage III or stage IV MM.^4^ The results showed no significant difference in efficacy between the two groups, but grade ≥3 TRAEs were significantly lower in the NIVO3 + IPI1 group (33.9% vs 48.3%, *p* = 0.006). In the KEYNOTE‐029 trial, IPI 50 mg/body (IPI 50) was administered every 6 weeks, or IPI 100 mg/body (IPI 100) every 12 weeks for four cycles in combination with pembrolizumab in patients with advanced‐stage MM.^14^ At the 16‐month follow‐up, grade 3–5 TRAEs were observed in 24% of the patients in the IPI 50 group and 39% in the IPI 100 group, with a significant reduction in toxicity in the IPI 50 group. The ORR was 55% for the IPI 50 group and 61% for the IPI 100 group, both meeting the threshold for comparison with conventional pembrolizumab alone. The results of the previous studies and the present study suggest that a dose of IPI lower than 3 mg/kg may reduce the risk of side effects when combined with anti‐PD‐1 therapy in advanced‐stage MM. However, further studies are needed to compare the efficacy of these treatments.

This study has some limitations. First, due to its retrospective nature, selection bias could not be excluded, and some data may have been missing or inaccurately collected. Second, the sample size, especially in the subgroup analyses, may be too small for adequate statistical evaluation. Third, a few patients in the NIVO+IPI ≤2 cycles group intentionally discontinued NIVO+IPI treatment after two cycles. Therefore, caution is needed when interpreting whether NIVO+IPI should be stopped after two cycles.

In conclusion, the optimal number of NIVO+IPI cycles in Japanese patients with advanced‐stage MM requires additional study, but an efficacy assessment after the second dose may reduce unnecessary NIVO+IPI administration. It is also suggested that reducing the IPI dose of NIVO+IPI from 3 mg/kg to 2 mg/kg may be more beneficial in Japanese patients with advanced MM, especially if side effects are a concern.

## CONFLICT OF INTEREST STATEMENT

None declared.

## ETHICS STATEMENT

This study was reviewed and approved by the Ethics Committee of Shizuoka Cancer Center (2024/9/19); approval number: J2024‐108.
